# Novel Scaffolds Fabricated Using Oleuropein for Bone Tissue Engineering

**DOI:** 10.1155/2014/652432

**Published:** 2014-05-13

**Authors:** Hui Fan, Junfeng Hui, Zhiguang Duan, Daidi Fan, Yu Mi, Jianjun Deng, Hui Li

**Affiliations:** ^1^Shaanxi Key Laboratory of Degradable Biomedical Materials, School of Chemical Engineering, Northwest University, 229 Taibai North Road, Xi'an 710069, China; ^2^Shaanxi R&D Center of Biomaterials and Fermentation Engineering, School of Chemical Engineering, Northwest University, Xi'an 710069, China

## Abstract

We investigated the feasibility of oleuropein as a cross-linking agent for fabricating three-dimensional (3D) porous composite scaffolds for bone tissue engineering. Human-like collagen (HLC) and nanohydroxyapatite (n-HAp) were used to fabricate the composite scaffold by way of cross-linking. The mechanical tests revealed superior properties for the cross-linked scaffolds compared to the uncross-linked scaffolds. The as-obtained composite scaffold had a 3D porous structure with pores ranging from 120 to 300 **μ**m and a porosity of 73.6 ± 2.3%. The cross-linked scaffolds were seeded with MC3T3-E1 Subclone 14 mouse osteoblasts. Fluorescence staining, the Cell Counting Kit-8 (CCK-8) assay, and scanning electron microscopy (SEM) indicated that the scaffolds enhanced cell adhesion and proliferation. Our results indicate the potential of these scaffolds for bone tissue engineering.

## 1. Introduction

The need for bone grafts to repair skeletal defects caused by trauma or bone neoplasia has been constantly increasing in recent years. Currently, autologous bone grafts and allografts are the main options for bone replacement. Although autologous bone grafts are ideal for osteoinduction and osteogenesis, they require secondary surgery, are available in very limited supplies, and can lead to donor site morbidity [1, 2]. In addition, allografts can potentially result in disease transmission and immune responses. All of these factors limit their application in bone reconstruction. To overcome these limitations, various bone tissue engineering strategies have been proposed. The ideal scaffold for use as a transplant not only has good biocompatibility, appropriate mechanical properties, and a well-matched degradation rate [3–5] but also has an appropriate pore size and high interconnectivity to promote cells attachment, proliferation, and bone repair [6–10].

To provide a biocompatible and bioactive environment for new bone formation, a wide variety of materials have been used to mimic the bone-forming components. As the main structural element in skin, bone, tendon, cartilage, blood vessels, and heart valves, collagen has been widely used in tissue engineering. Human-like collagen (HLC) is a recombinant collagen expressed by recombinant* Escherichia coli *BL21 [11], which contains a modified cDNA reverse-transcribed from human collagen mRNA. Due to the water solubility, workability, low immunogenicity, biocompatibility, and biodegradability of HLC, this material has been successfully used for vascular scaffolds [12, 13], artificial bone [14], hydrogels [15], and skin tissues [16]. As the major components of human natural bone, nanohydroxyapatite (n-HAp) possesses excellent biocompatibility, osteoconductivity, and bioactivity and lacks antigenicity and cytotoxicity. Thus, n-HAp is an outstanding biomaterial for guided bone regeneration [17, 18].

In addition, the cross-linking technique can increase the mechanical properties of the scaffolds. Chemical cross-linking agents, such as carbodiimide or glutaraldehyde, have been studied extensively for biomedical applications. However, their high cytotoxicity may influence the biocompatibility of the scaffolds [19]. Thus, the development of a natural noncytotoxic cross-linking agent is urgently needed. Simple phenolic compounds derived from plants have been studied for cross-linking proteins [20]. However, little attention has been focused on polyphenol as a cross-linking agent for bone tissue engineering. Furthermore, oleuropein, a polyphenol belonging to the secoiridoid class, the most representative catecholic components of olives, possesses high antioxidation ability due to its ability to scavenge superoxide radicals. Antioxidant nutrients might reduce the production of free radicals, contributing to bone resorption and enhancing bone formation. It has been demonstrated that oleuropein elicits protective effects on bone [21].

In this study, oleuropein was employed as a cross-linking agent for bone tissue engineering using cross-linked composite HLC/n-HAp scaffolds. The characteristics and mechanical properties of the scaffolds and their ability to promote cell adhesion and proliferation were investigated.

## 2. Materials and Methods

### 2.1. Materials

HLC was supplied from Juzi Biogene Technology Co. Ltd. (97,000 Da, Xi'an, China) and n-HAp was supplied by Epri Nano Materials Ltd. Co. (20 nm, Nanjing, China). Oleuropein was supplied by Wedar Ltd. Co. (Shanghai, China). MC3T3-E1 cells were obtained from Biok&KM Co. Ltd. (Jiangsu, China). Trypsin (250 units/mg) was obtained from Amresco (Solon, OH, USA). Minimum essential medium (MEM) and fetal bovine serum (FBS) were purchased from Hyclone (Logan, UT, USA). The Cell Counting Kit-8 (CCK-8) was obtained from Keygen Biological Technology Development Co. Ltd. (KGA317, Nanjing, China). All other reagents and solvents were of analytical grade.

### 2.2. Preparation of Composite HLC/n-HAp Scaffolds

HLC was dissolved in deionized distilled water at a concentration of 4.8% (w/v) by gentle stirring at room temperature for 30 min. The n-HAp (HLC/n-HAp ratio = 1 : 2, 1 : 3, 1 : 4, 1 : 5, and 1 : 6 (w/w)) was then dispersed in the HLC solution. The HLC/n-HAp mixture was transferred into a mold, which was successively frozen at 4°C for 20 min, −20°C for 1 h, and −70°C for 3 h. After lyophilization in a vacuum freeze-dryer (FD 5–10, SIM, USA) for 48 h at 6.7–13.3 Pa (50–100 mTorr), the as-obtained scaffolds were cross-linked using an oleuropein ethanol-water solution (concentration of 0.5%, 1%, 1.5%, 2%, 2.5%, and 3% (w/v) and 90% ethanol) at 37°C for 36 h. The scaffolds were then washed under running deionized distilled water for 3 h to remove the ethanol and again lyophilized for 48 h. The scaffolds were applied to subsequent experiments.

### 2.3. Scanning Electron Microscopy (SEM)

The surface morphologies of the composite HLC/n-HAp scaffolds were examined by scanning electron microscopy (Hitachi S-570, Japan). Before imaging, the scaffolds were cut into pieces with a razor blade, which were mounted on aluminum stubs and sputter-coated with gold.

### 2.4. Mechanical Properties of the HLC/n-HAp Scaffolds

The mechanical property of the composite HLC/n-HAp scaffolds was determined by measurement of the compression strength and Young's modulus using an INSTRON 5565 Materials Testing System with a 5000 N load cell. To test the longitudinal compression strength, the loading rate was 1 mm/min. Five samples were measured for each group. Cylindrical samples were prepared with diameters of 10 mm and lengths of 30 mm.

### 2.5. X-Ray Diffraction (XRD)

The crystalline phase of the scaffold was analyzed by X-ray diffraction (Rigaku D/max-3C, Japan). The phases were identified by comparison to the n-HAp X-ray diffractograms. The scaffold was ground to a powder and analyzed. The XRD data were acquired using a voltage of 40 kV at a rate of 2°/min and angle range of 10–60°.

### 2.6. Fourier Transform Infrared Spectra (FTIR)

The chemical structures of the composite HLC/n-HAp scaffold, HLC, and n-HAp were characterized using a Fourier transform infrared spectrophotometer (EQUINOX-55, Bruker Corporation, Germany) using the KBr method. FTIR spectra were collected from 4000 to 500 cm^−1^.

### 2.7. Thermogravimetric Analysis (TGA)

The thermal stability of the composite scaffolds was evaluated by thermogravimetric analysis (STA449C, Netzsch) with a heating rate of 3°C/min from 30°C to 600°C.

### 2.8. Scaffold Porosity

The porosity of the scaffold was measured by liquid displacement, as calculated according to *P* = (*W*_1_ − *W*_0_)/*ρV*_0_, where *W*_1_ is the wet weight of the scaffold after it is immersed in the dehydrated alcohol for 48 h until it is saturated, *W*_0_ is the dry weight of the scaffold, *ρ* is the density of the dehydrated alcohol, and *V*_0_ is the volume of the scaffold. Three parallel samples were tested.

### 2.9. Cell Seeding and Culture

MC3T3-E1 Subclone 14 mouse osteoblasts were used to evaluate cell proliferation and morphology on the scaffolds. MC3T3-E1 cells were cultured in minimum essential medium (MEM) including 10% heat-inactivated FBS in a 95% relative humidity atmosphere of 5% CO_2_ at 37°C. The composite scaffolds were cut into circular disks of 10 mm diameter and 3 mm height. The pieces were placed in 48-well culture plates and sterilized by Co_60_ irradiation. Before cell seeding, the scaffolds were prewetted with MEM for 24 h to displace air from the scaffolds. The MC3T3-E1 cells were digested by trypsin/EDTA solution and suspended in MEM at a concentration of 1 × 10^6^ cell/mL. A total of 50 *μ*L of cell suspension was seeded onto each scaffold surface. The seeded scaffolds were incubated at 37°C with 5% CO_2_ to allow cells to attach. After 4 h, an additional 1 mL of medium was added to each well. The culture medium was refreshed every two days. After 3, 7, and 14 days, the cell-scaffold constructs were analyzed.

### 2.10. Cell Viability and Proliferation

Evaluation of the cell viability and proliferation on the scaffolds was performed by fluorescence staining and the CCK-8 assay. For fluorescence staining, each cell-scaffold was carefully washed with PBS and the nuclei were stained with 4′,6-diamidino-2-phenylindole (DAPI) solution (1 : 1000 DAPI in PBS) for 10 min. The cell-scaffolds were analyzed using a fluorescence microscope (TS100, Nikon, Japan). For the CCK-8 assay, the cell-seeded scaffolds were transferred to new 48-well plates and incubated with 1 mL fresh MEM medium containing 10 *μ*L CCK-8 at 37°C with 5% CO_2_ for 3 h. An unseeded scaffold was used as a control. A total of 100 *μ*L of reaction liquid was transferred to a 96-well plate to measure the absorbance at 450 nm using a microplate reader (Power Wave XS2, Gene Company, USA). Scaffolds with medium but without cells were used to assess the background absorbance. The degree of cell proliferation was determined after 3, 7, and 14 days of culture. Five cell-scaffold constructs were tested each time.

### 2.11. Morphological Analysis

Scanning electron microscopy was used to observe the morphology of cells adhered to the scaffolds. After 7 and 14 days of culture, the cell-seeded scaffolds were rinsed with PBS and fixed with 2.5% glutaraldehyde in phosphate buffer (pH 7.4) at 4°C for 4 h. The scaffolds were dehydrated in increasing concentrations of ethanol (30%, 50%, 70%, 90%, 95%, and 100%) and critically point-dried. The dried cell-scaffolds were mounted on aluminum stubs and sputter-coated with gold for SEM.

### 2.12. Statistical Analysis

The data were analyzed using the Statistical Analysis System (SAS 9.0) package software for analysis of variance using Duncan's test. All experiments were carried out in triplicate. Significance was established at *P* ≤ 0.05.

## 3. Results

### 3.1. Effects of Processing Parameters on the Properties of Scaffolds

**Figure 1:**
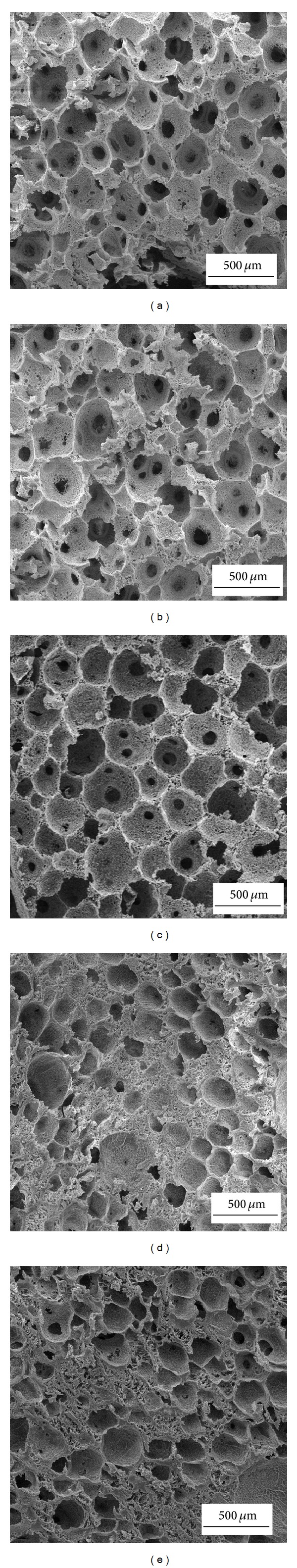
SEM images of different samples. The ratio of HLC/n-HAp is 1 : 2 (a), 1 : 3 (b), 1 : 4 (c), 1 : 5 (d), and 1 : 6 (e).

**Figure 2:**
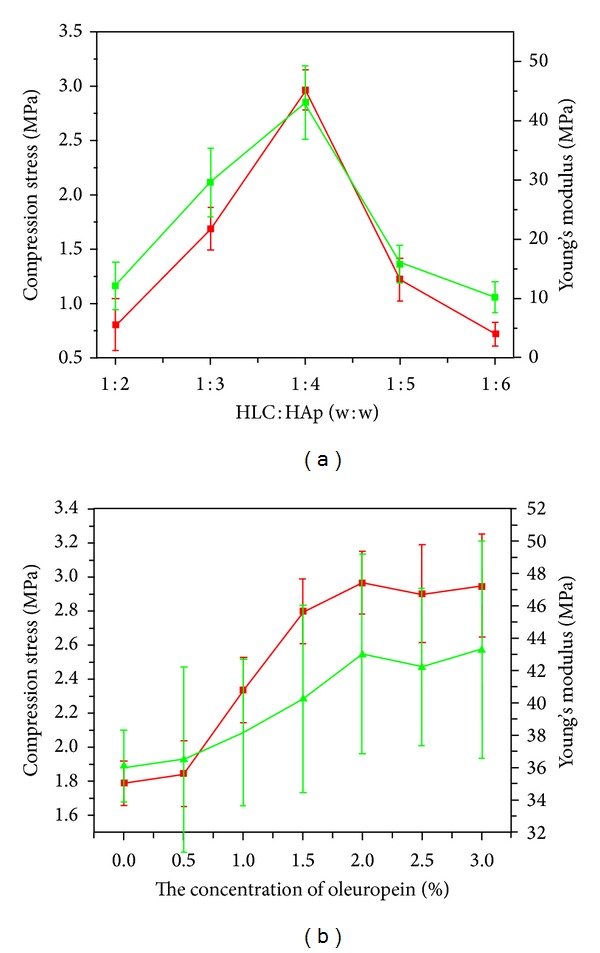
Compressive strength (red) and Young's modulus (green) for the different samples. (a) The composite scaffolds were formed with different n-HAp contents using a 2% solution of oleuropein. (b) The scaffolds were formed with different concentration of oleuropein at an HlC/n-HAp ratio of 1 : 4.

The porosity for the different samples is reported in Table 1. With the ratio of HLC to n-HAp decreasing from 1 : 2 to 1 : 6, the porosity of the scaffolds decreased from approximately 89.3 ± 4.1% to 51.57 ± 1.5%. This was coincident with the SEM images of the different samples, as shown in Figure 1. The samples with ratios of 1 : 2, 1 : 3, and 1 : 4 had homogeneous pores that were interconnected. The samples with ratios of 1 : 5 and 1 : 6 had weakly interconnected pores that were inhomogeneous. The effects of different composites and the oleuropein concentration on the compressive strength and Young's modulus were investigated. As shown in Figure 2(a), increasing the n-HAp content of the scaffolds to 80 wt.% increased the compressive strength and Young's modulus to 2.97 ± 0.19 MPa and 43.03 ± 6.17 MPa, respectively. When the n-HAp content of the scaffolds was more than 80 wt.%, the brittleness of the scaffolds increased significantly. As shown in Figure 2(b), when the concentration of oleuropein solution reached 2% (w/v), the compressive strength and Young's modulus reached maximums of 2.97 ± 0.19 MPa and 43.03 ± 6.17 MPa, respectively. The composite scaffold with a porosity of 73.6 ± 2.3 and a compressive strength of 2.97 ± 0.19 was used for subsequent characterization and cell culture. The effect of the sterilization procedure by Co_60_ irradiation on the HLC/n-HAp scaffold (HLC : n-HAp = 1 : 4, with concentration of oleuropein being 2%) was also investigated.

### 3.2. Morphology of the HLC/n-HAp Composite Scaffolds

**Figure 3:**
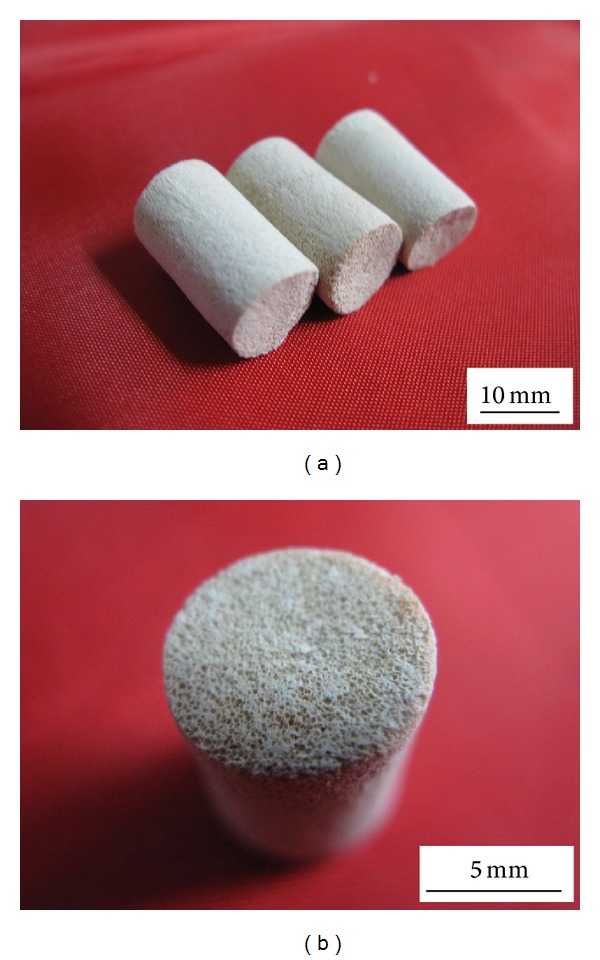
Images of (a) HLC/n-HAp scaffolds and (b) cross-sections with homogeneous pores.

**Figure 4:**
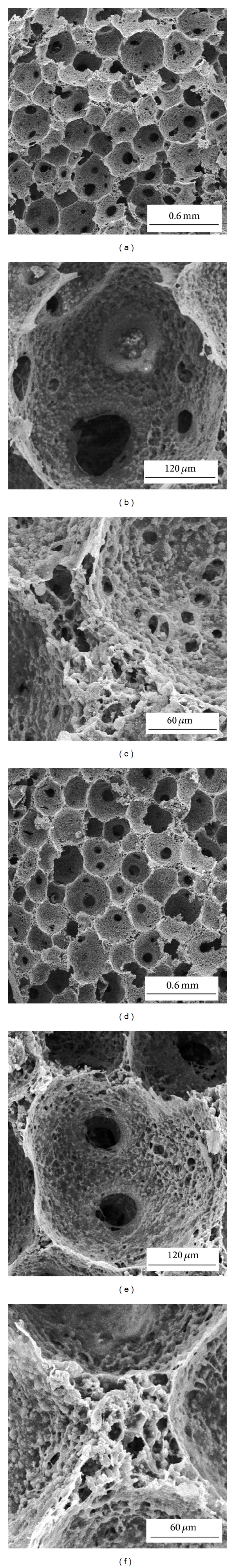
SEM images of the cross-sectional surfaces of the HLC/n-HAp scaffolds before ((a), (b), and (c)) and after ((d), (e), and (f)) Co_60_ irradiation. ((a), (d)) The scaffolds display interconnected pores ranging in size from 120 to 300 *μ*m. ((b), (e)) A typical macroporous microstructure for the scaffolds. ((c), (f)) The pore walls in the scaffold.

The macroscopic view and the morphology of the HLC/n-HAp scaffolds are shown in Figures 3 and 4, respectively. The scaffolds (before and after Co_60_ irradiation) had 3D porous structures with homogeneous pores ranging from 120 to 300 *μ*m and the porosity was 73.6 ± 2.3%. The pores were interconnected, which might be helpful for water and nutrient transport. On the walls of the macropores there were smaller pores, with pore sizes less than 6 *μ*m. The Co_60_ irradiation sterilization procedure did not have any obvious influence on the scaffold morphology.

### 3.3. Characterization of the HLC/n-HAp Composite Scaffolds

**Figure 5:**
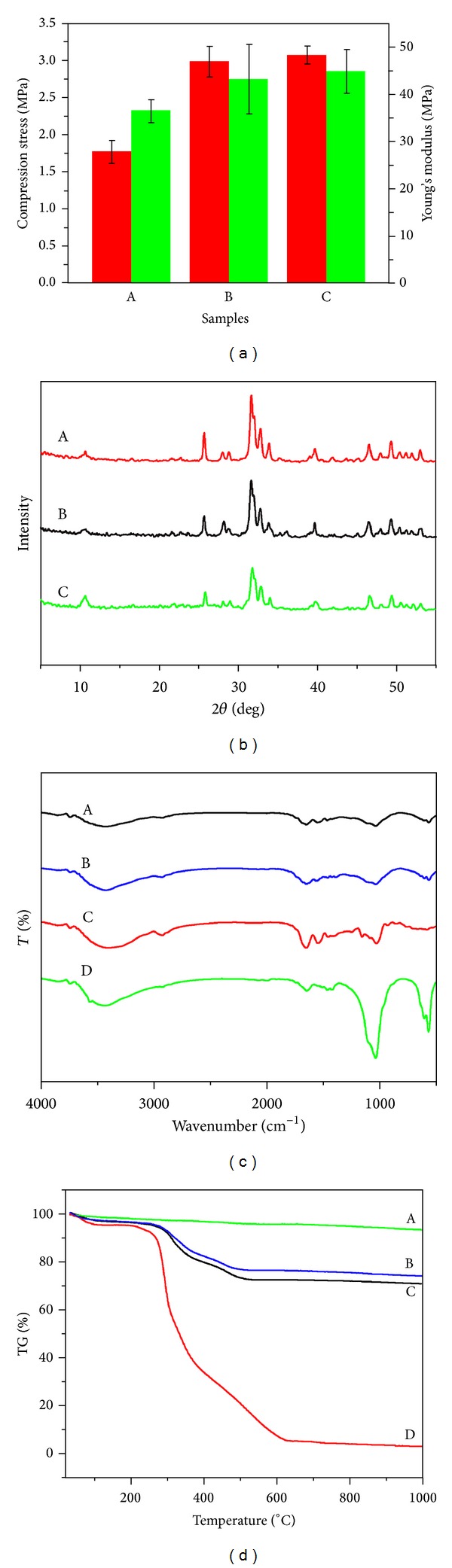
HLC/n-HAp scaffolds characterization. (a) Compressive strength (in red) and Young's modulus (in green) of the scaffolds (A) after and (B) before the cross-linking procedure and (B) before and (C) after Co_60_ irradiation. (b) XRD patterns for (A) the n-HAp, HLC/n-HAp scaffolds (B) before and (C) after Co_60_ irradiation. (c) FT-IR spectra for the HLC/n-HAp scaffolds (A) before, (B) after Co_60_ irradiation, (C) for HLC, and (D) for n-HAp. (d) TGA curves for the (A) n-HAp, HLC/n-HAp scaffolds (B) after, (C) before Co_60_ irradiation, and (D) for HLC.

Compression tests were conducted to assess the mechanical performance of the scaffolds. The compressive strength of the HLC/n-HAp scaffolds was enhanced by cross-linking, which indicated that the cross-linking process contributed to the superior mechanical properties of the scaffolds.  Figure 5(a) shows the mechanical properties of the scaffolds before and after the sterilization by Co_60_ irradiation. There was no obvious difference in the mechanical properties of the scaffold after sterilization.

As shown in Figure 5(b), the composite scaffolds displayed sharp and intense diffraction peaks at 25.8°, 31.8°, 33°, 34°, 39.8°, 46.7°, and 49.4°, which confirmed the presence of hydroxyapatite.

Figure 5(c) shows the FT-IR spectra of the scaffolds (before and after Co_60_ irradiation), along with the spectra for n-HAp and HLC. In the n-HAp spectra, an absorption band associated with the –OH stretching vibration mode is clearly seen at 3436 cm^−1^. The peaks at approximately 1039, 602, and 567 cm^−1^ were assigned to PO_4_^3−^. The characteristic absorption peaks of HLC were 3429, 1650, and 1237 cm^−1^, which were attributed to the N–H stretching vibration peaks, C=O peaks, and the combined peaks between the C–N stretching vibration and N–H bending vibration, respectively. These characteristic absorption peaks were also found in the spectra of the composite scaffolds. The scaffolds showed the same characteristic absorption peaks before and after Co_60_ irradiation.

As observed from the TGA curve (Figure 5(d)), there were two main decreases in the mass of the HLC and HLC/n-HAp scaffolds (before and after Co_60_ irradiation). The n-HAp did not have any obvious weight loss. The weight loss for the HLC and the scaffolds below 120°C could be attributed to the loss of freely bound water. From 250°C to 600°C, the significant decrease in the mass could be attributed to the decomposition of HLC. Thermal degradation of HLC began at 250°C, while the onset of thermal degradation for the scaffolds was approximately 300°C. The electrostatic interaction between HLC and n-HAp, as well as the cross-linking procedure, resulted in an altered thermal degradation profile. The Co_60_ irradiation had no obvious influence on the thermal stability of the scaffolds.

### 3.4. Cell Viability and Proliferation

**Figure 6:**
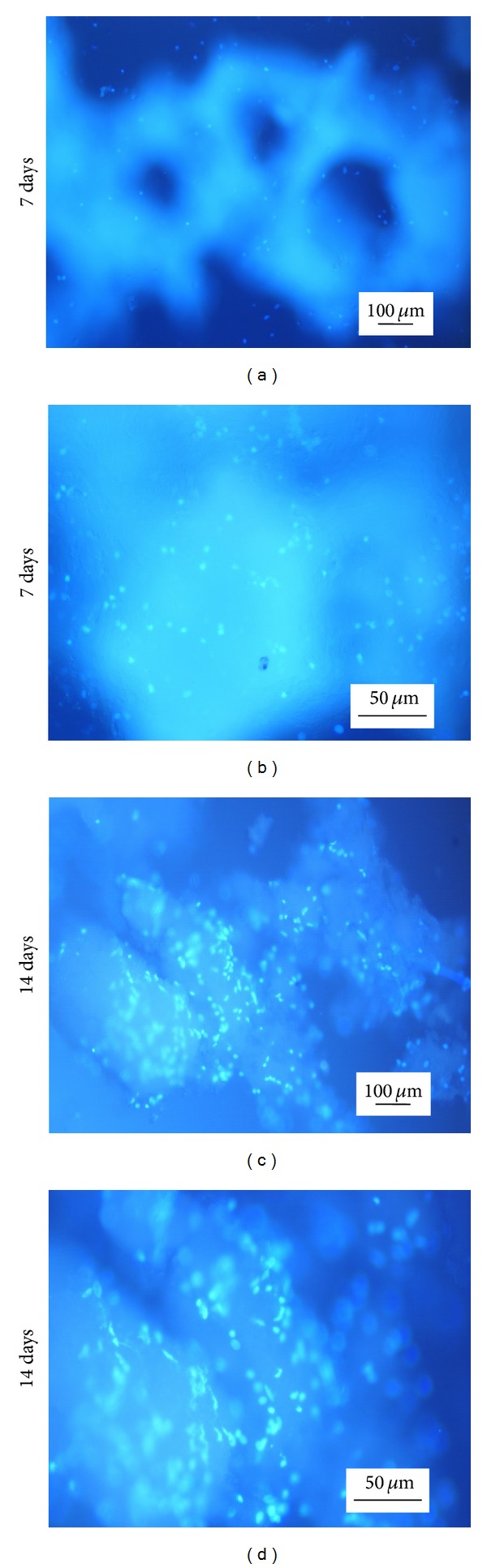
Fluorescence images of MC3T3-E1 cells cultured on the HLC/n-HAp scaffolds for ((a), (b)) 7 days and ((c), (d)) 14 days. ((a), (c)) Low magnification views of the cell-scaffolds. ((b), (d)) Magnified views of the cells on the pore walls of the scaffolds. The scale bars in the left column and right column represent 100 *μ*m and 50 *μ*m, respectively.

**Figure 7:**
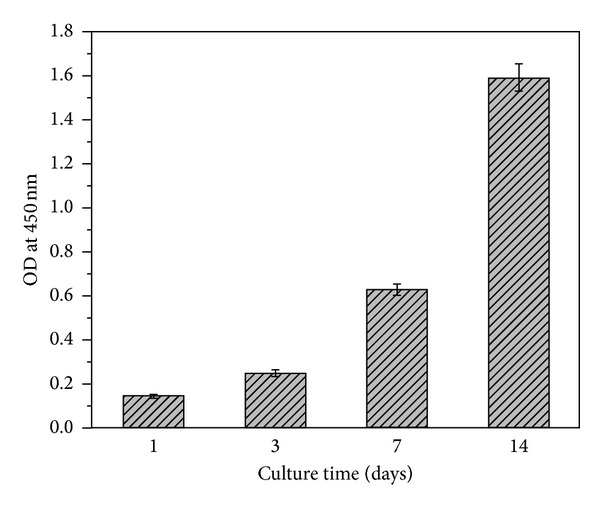
CCK-8 assay for attachment and proliferation of MC3T3-E1 cells on the HLC/n-HAp scaffolds over various incubation periods. Error bars represent means ± SD (*n* = 5).

We evaluated the viability and proliferation of MC3T3-E1 cells on the as-prepared scaffolds.  Figure 6 shows fluorescence images of the cell-scaffolds incubated for 7 and 14 days. With increasing culture time, more area was covered by the cells. It was clear that the MC3T3-E1 cells adhered and proliferated well. As shown in Figure 7, the CCK-8 assay revealed that the absorbance values increased significantly with culture time. This suggested that the pores and the surfaces of the scaffolds enhanced cell adhesion and proliferation. These results further indicate that the HLC/n-HAp scaffolds are nontoxic and biocompatible.

### 3.5. Cell Morphology

**Figure 8:**
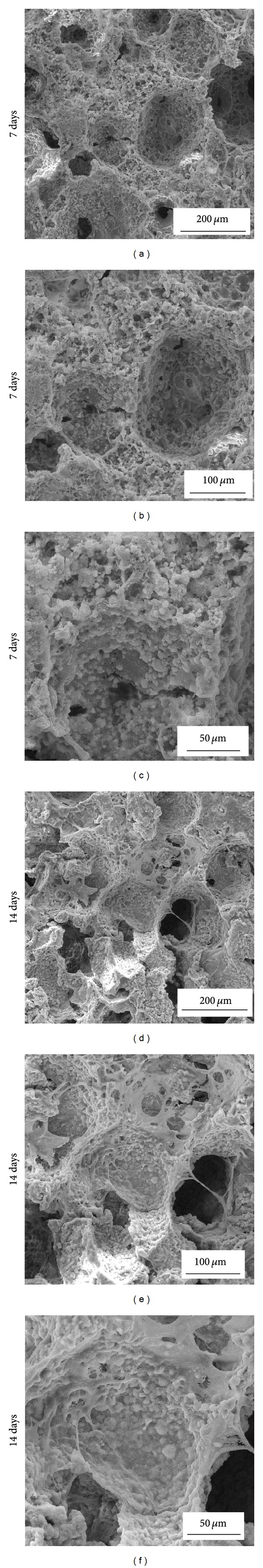
SEM images of MC3T3-E1 cells cultured on the HLC/n-HAp scaffolds for 7 ((a), (b), and (c)) and 14 ((d), (e), and (f)) days. The scale bars in the left column, middle column, and right column represent 200 *μ*m, 100 *μ*m, and 50 *μ*m, respectively.

SEM images of the cells grown on the scaffolds are shown in Figure 8. A greater number of MC3T3-E1 cells were observed to attach, spread, and proliferate at 7 and 14 days on the scaffolds. After 7 days of incubation, almost all of the scaffold surfaces were covered with spherical cells. The cells extended more cellular protrusions and connected with each other by way of these structures. After 14 days in culture, the cells produced a large amount of extracellular matrix (ECM), in which the cells were embedded.

## 4. Discussion

A new strategy to fabricate biodegradable scaffolds with more than two components with different physicochemical properties was proposed for bone tissue engineering, when a one-component scaffold cannot suffice [22, 23]. In this study, an HLC/n-HAp scaffold was fabricated, which preserved the biological characteristics of the HLC and the mechanical properties and osteoconductivity of the n-HAp. We fabricated highly porous 3D structure scaffolds with homogeneous and interconnected pores by slow cooling and vacuum freeze-drying. These water-soluble scaffolds were transformed into water-insoluble scaffolds using the natural cross-linking agent oleuropein. After cross-linking, another freeze-drying step was performed.

In bone tissue engineering, it is important to have the appropriate pore sizes for cell adhesion and tissue reconstruction. The pore size greatly affects the cellular activity. Even subtle changes in pore size may have significant effects on cell adhesion [24]. It has been reported that small pore sizes can limit cell migration and colonization [25, 26], vascular ingrowth, and nutrient and water transfer [27]. Furthermore, smaller pore sizes can influence the cell distribution in the scaffolds. However, if the pore size is too large, it can influence cell adhesion. Large pore sizes affect the construct and mechanical properties of the scaffolds. Moreover, it is difficult to create a suitable environment for the production of ECM in scaffolds with large pore sizes [28]. Freeze-drying has proved to be a gentle drying method that can be used to obtain scaffolds with appropriate pore size and interconnectivity [29]. As shown in Figures 3(b) and 4, the pores of the HLC/n-HAp scaffolds are homogeneous and interconnected. The SEM images (Figure 8) indicate that cells are contained within the scaffold pores, forming bridges over the pores (Figure 8(e)). The cells connected with neighboring cells by way of the pore structures. The HLC/n-HAp scaffolds promoted MC3T3-E1 ECM production, which is essential for bone formation.

An ideal tissue engineering scaffold provides sufficient mechanical properties and high porosity, both of which are key determinant factors for implantation. Proper mechanical properties and porosity are needed to supply temporary support for vascularization and tissue ingrowth [30]. However, exceedingly high porosity can reduce the mechanical property of the scaffolds. The compressive strength is also closely tied to the proportion of inorganic and organic ingredients [31]. In this case, an HLC and n-HAp ratio of 1 : 4 was employed to obtain a better scaffold with a porosity of 73.6 ± 2.3% and a mechanical strength of 3 MPa. In addition, the cross-linking process further contributed to the mechanical properties, as shown in Figure 5(a).

The rate and quality of new tissue formation are greatly affected by the initial cell adhesion to the scaffolds. Cell adhesion is influenced by the scaffold surface characteristics, such as the surface chemical composition, surface topography, surface multicavities, and roughness [32]. Higher initial cell adhesion requires adhesion proteins that are abundant in the serum. The n-HAp nanoscale crystals have a high binding affinity to the serum proteins [33]. When a higher degree of proteins is absorbed on the scaffold surface, it could provide more attachment sites for the cells. In this study, the use of nanoscale n-HAp greatly promoted cell adhesion. In addition, the surface roughness (Figures 4(c) and 4(f)) could improve cell-biomaterial response, adding cellular adhesion, growth, migration, and differentiation. The cell spreading and proliferation significantly increase on rough surfaces, compared to smoother surfaces [32]. As shown in the SEM images, the cells established close contact with the scaffold surface, displaying spherical morphologies (Figures 8(c) and 8(f)) with numerous filopodia and lamellipodia (Figures 8(d), 8(e), and 8(f)).

Cytotoxicity must be evaluated for all scaffolds used in bone tissue engineering. In this study, the natural cross-linking agent oleuropein was used. The most common cross-linking agents, glutaraldehyde and carbodiimide, are cytotoxic and may negatively affect the cells [34]. In our study, the natural cross-linking agent, oleuropein, was assessed using the CCK-8 assay. As shown in Figure 7, the composite HLC/n-HAp scaffolds are biocompatible and can thus serve as suitable materials for biomedical applications.

## 5. Conclusion

A composite HLC/n-HAp system with interconnected pores was prepared by cross-linking. The as-prepared scaffolds were characterized using SEM, XRD, FTIR, and TGA. In addition, the porosity, compressive strength, cytotoxicity, cell adhesion, and proliferation of the scaffolds were investigated. The scaffolds had interconnected pores ranging from 120 to 300 *μ*m. The cells attached well to the pore walls, extending cellular protrusions and producing large amounts of ECM. The HLC/n-HAp scaffolds were noncytotoxic and biocompatible. The scaffolds preserved the outstanding biological characteristics of HLC and the excellent osteoconductivity of n-HAp. Therefore, the HLC/n-HAp scaffolds obtained by oleuropein cross-linking have potential for use in bone tissue engineering.

## Figures and Tables

**Table 1:** Porosity of the composite scaffolds with different n-HAp contents.

HLC : n-HAp ratio (w/w)	Porosity (%)
1 : 2	89.3 ± 4.1
1 : 3	81.2 ± 2.8
1 : 4	73.6 ± 2.3
1 : 5	60.8 ± 2.0
1 : 6	51.6 ± 1.5
